# Network Analysis of Depressive Symptoms Among Residents of Wuhan in the Later Stage of the COVID-19 Pandemic

**DOI:** 10.3389/fpsyt.2021.735973

**Published:** 2021-09-30

**Authors:** Na Zhao, Wen Li, Shu-Fang Zhang, Bing Xiang Yang, Sha Sha, Teris Cheung, Todd Jackson, Yu-Feng Zang, Yu-Tao Xiang

**Affiliations:** ^1^Unit of Psychiatry, Department of Public Health and Medicinal Administration, and Institute of Translational Medicine, Faculty of Health Sciences, University of Macau, Macao, SAR China; ^2^Center for Cognition and Brain Disorders, The Affiliated Hospital of Hangzhou Normal University, Hangzhou Normal University, Hangzhou, China; ^3^Institute of Psychological Sciences, Hangzhou Normal University, Hangzhou, China; ^4^Shanghai Key Laboratory of Forensic Medicine, Key Laboratory of Forensic Science, Ministry of Justice, Shanghai Forensic Service Platform, Academy of Forensic Science, Shanghai, China; ^5^Research Center for Psychological and Health Sciences, China University of Geosciences, Wuhan, China; ^6^Department of Psychiatry, Wuhan Mental Health Center, Wuhan, China; ^7^School of Health Sciences, Wuhan University, Wuhan, China; ^8^The National Clinical Research Center for Mental Disorders & Beijing Key Laboratory of Mental Disorders, Beijing Anding Hospital and the Advanced Innovation Center for Human Brain Protection, Capital Medical University, School of Mental Health, Beijing, China; ^9^School of Nursing, Hong Kong Polytechnic University, Hong Kong, Hong Kong, SAR China; ^10^Department of Psychology, University of Macau, Macao, SAR China; ^11^Centre for Cognitive and Brain Sciences, University of Macau, Macao, SAR China; ^12^Institute of Advanced Studies in Humanities and Social Sciences, University of Macau, Macao, SAR China

**Keywords:** depression, network analysis, COVID-19, Wuhan, mental health

## Abstract

**Background:** Depression has been a common mental health problem during the COVID-19 epidemic. From a network perspective, depression can be conceptualized as the result of mutual interactions among individual symptoms, an approach that may elucidate the structure and mechanisms underlying this disorder. This study aimed to examine the structure of depression among residents in Wuhan, the epicenter of the COVID-19 outbreak in China, in the later stage of the COVID-19 pandemic.

**Methods:** A total of 2,515 participants were recruited from the community via snowball sampling. The Patient Health Questionnaire was used to assess self-reported depressive symptoms with the QuestionnaireStar program. The network structure and relevant centrality indices of depression were examined in this sample.

**Results:** Network analysis revealed Fatigue, Sad mood, Guilt and Motor disturbances as the most central symptoms, while Suicide and Sleep problems had the lowest centrality. No significant differences were found between women and men regarding network structure (maximum difference = 0.11, *p* = 0.44) and global strength (global strength difference = 0.04; female vs. male: 3.78 vs. 3.83, *p* = 0.51), a finding that suggests there are no gender differences in the structure or centrality of depressive symptoms.

**Limitations:** Due to the cross-sectional study design, causal relationships between these depressive symptoms or dynamic changes in networks over time could not be established.

**Conclusions:** Fatigue, Sad mood, Guilt, and Motor disturbances should be prioritized as targets in interventions and prevention efforts to reduce depression among residents in Wuhan, in the later stage of the COVID-19 pandemic.

## Introduction

Coronavirus disease 2019 (COVID-19) was first found in Wuhan, China and was subsequently reported in over 200 countries and territories. The virus, together with quarantine and isolation measures (1–3), contributed to increases in common psychiatric syndromes such as anxiety and depression (3–9). Based on previous findings (10, 11), psychiatric syndromes of infectious diseases (e.g., Influenza and Ebola), particularly depression, can persist long after the peak of an outbreak. Of the psychiatric disorders that increase during and after pandemics, depression is among the most common and debilitating syndromes, and is associated with a range of negative health outcomes including cognitive impairments (12), increased risk of suicide (13) and cardiovascular disease risk (14), high disease burden and lowered quality of life (15, 16). For instance, one study found that 50.4% of people who were exposed to COVID-19 patients experienced depressive symptoms (17) while the corresponding figure was 20.4% in the general population in China during the COVID-19 epidemic (2). A meta-analysis of 12 studies (18) revealed that the prevalence of depressive symptoms was 25% during the COVID-19 outbreak.

Traditional theories of psychopathology assume individual symptoms of a particular psychiatric disorder are the manifestations of a latent variable but do not explore how these symptoms interact with each other (19, 20). In traditional frameworks, for example, depression is the common cause of a collection of symptoms, such as sad mood, fatigue, and insomnia (21, 22). As symptoms are the indicators of an underlying disorder, instruments with a set of items, such as Patient Health Questionnaire (PHQ-9) and the Beck Depression Inventory (BDI), are commonly used to investigate whether or not an individual suffers from depression. In traditional perspectives, individual symptoms are interchangeable and are not distinct from each other in their mechanisms or impact on functional impairment (23). Furthermore, individual symptoms in traditional models usually share a common origin, although, in fact, some symptoms are more strongly associated with other symptoms (24), and also impair different functions (23).

In recent years, network analysis (NA) has been proposed as a novel alternative approach to conceptualizing psychiatric disorders. From an NA perspective, a psychiatric disorder consists of a set of dynamically interacting, reciprocally reinforcing symptoms (20, 21). According to NA, depression is the result of interactions between a set of individual symptoms (20, 21, 25). For example, sleep problems may lead to fatigue, which, in turn, leads to motor problems or impaired concentration in depressed patients. In the depression symptom network, central symptoms feature the most connections with other symptoms and can also trigger other symptoms. As such, pinpointing central symptoms has important clinical implications for developing effective targeted strategies or interventions to treat psychiatric disorders (21, 25, 26).

Because infectious disease epidemics contribute to the rise and persistence of psychiatric disorders, particularly depression (27) in the general population (10, 11), it is important to examine the structure of depression in the context of groups most directly affected by an epidemic. In China, the COVID-19 outbreak had been well-controlled by the middle of 2020, although there continued to be some imported cases from time to time (28–30). Although one study using NA examined changes in depression and anxiety symptoms during the peak of the COVID-19 pandemic (5), no NAs have been published on the aftermath of the COVID-19 peak, although it is important to develop timely treatment and preventive measures for depression in stages after the peak of the COVID-19 pandemic has receded. Therefore, we aimed to explore associations of individual depression symptoms in Wuhan residents in the later stage of the COVID-19 pandemic.

## Methods and Materials

### Participants

This cross-sectional study was conducted in Wuhan between 25 May and 13 June 2020 using snowball sampling. The assessment was conducted using the QuestionnaireStar program, which has been widely used in epidemiological studies (31–33). To be eligible for this study, participants needed to meet the following criteria: [1] aged 18 years or above; [2] current residents of Wuhan who could be able to read Chinese and understand the contents of the assessments; [3] not infected with COVID-19 during the pandemic. All participants were required to electronically sign the written informed consent. The study protocol was approved by the ethics committee of Beijing Anding Hospital (2020-Keyan; No. 10).

### Data Collection

Basic demographic data were collected. The Chinese version of the Patient Health Questionnaire (PHQ-9) was used to measure depression symptoms (34). The PHQ-9 consists of 9 items investigating depressive symptoms, including Anhedonia, Sad mood, Sleep problems, Fatigue, Appetite problems, Guilt, Impaired concentration, Motor disturbances and Suicidal ideation (35). Each item is scored from 0 (not at all) to 3 (nearly every day), with higher scores reflecting more severe depressive symptoms. The PHQ-9 had satisfactory psychometric properties (e.g., Cronbach's alpha coefficient = 0.86; 2-week test-retest reliability of *r* = 0.86; sensitivity and specificity of 0.86, respectively; convergent validity with other measures of depression and reduced well-being) in Chinese populations (36).

### Network Estimation

All analyses were conducted using R program (version: 4.0.3). Means, standard deviations (SD), kurtosis, and skewness of all PHQ-9 items were calculated. The informativeness of each symptom was estimated by means of SDs and possible item redundancy was checked using the R package *networktools* (22, 37, 38). Following previous studies (22, 37), for any two individual depressive symptoms, e.g., symptoms “A” and “B,” a correlation difference test was performed between the “A” correlation matrix (the correlations between symptom “A” and the remaining symptoms) and “B” correlation matrix (the correlations between symptom “B” and the remaining symptoms). If the proportion of significant differences between the “A” correlation matrix and “B” correlation matrix was <25% for all correlations, then symptoms “A” and “B” were classified as redundant.

All PHQ-9 item score distributions were skewed. Hence, following previous studies (38, 39), an Ising model was used to estimate the network. In the Ising model, all PHQ-9 items were dichotomized, with “0” and “1” representing the absence and presence of depressive symptoms, respectively. All item scores of “0” were considered to indicate the absence of a depressive symptom, while “1,” “2,” and “3” scores were considered to reflect the presence of a depressive symptom.

Network models consist of nodes and edges. Specifically, individual symptoms measured by the PHQ-9 represent “nodes,” whereas connections between nodes are “edges.” The Ising model assesses network structures after controlling for all the other associations between nodes in the network. Specifically, the Isling model identified relationships between nodes based on a Goodness-of-Fit measure, i.e., the least absolute shrinkage and selection operator (LASSO) with the extended Bayesian Information Criterion (EBIC) (eLASSO) (39, 40). This procedure can shrink weak connections to zero, and then reduce spurious associations, making the network interpretable (40). The R package *qgraph* was used for network visualization; the width and saturation of edges indicated the strength of association between each pair of nodes, while different colors indicated the direction of these correlations (i.e., the color green indicated positive correlations while the color red indicated negative correlations between each pair of nodes) (41).

Given controversies in the optimal method of modeling trichotomous items (21), we followed a recent published study (22) by adopting the EBIC graphical LASSO (EBICglasso) model to estimate the network (42). Similar to Ising models, EBICglasso models estimate partial polychoric correlations between any two given nodes. The network model was regularized using graphical LASSO based on EBIC, resulting in an interpretable network. Previous studies found that age, gender, marital status and education are often associated with depression (43–46). Therefore, as recommended by Dalege et al. (47), we re-estimated the initial network model after controlling for age, gender, marital status and education using R package *mgm*.

### Node Centralities

Three centrality indices (i.e., strength, betweenness and closeness) are often used to identify which symptoms are the most critical nodes (48, 49). Nevertheless, increasing evidence indicates that neither betweenness nor closeness is reliable in NA (50, 51). Hence, only strength (i.e., the sum values of absolute edge weights of a given node to all the other nodes) (25, 48), the most straightforward and frequently used centrality index (48), was calculated in this study. All analyses were performed using R package *bootnet and qgraph*.

### Network Accuracy and Stability

To examine the robustness of the estimated network, we assessed the accuracy of edge weights and node strength stability (42). The accuracy of edge weights was tested by constructing confidence intervals (CIs) with a 95% probability using non-parametric 1,000 times bootstrapping (25). Smaller and larger CIs signified more and less accurate edge weights, respectively.

Stability was assessed by using a case-dropping bootstrap method. Next, centrality indexes (i.e., strengths) in the subset sample (i.e., after removing certain cases) were compared with those from the overall sample (42, 52). Specifically, the correlation stability coefficient (*CS*-coefficient) was used to measure strength stability based on the maximum proportion of cases that can be dropped while maintaining the correlation of the ranking between original and subset networks at 0.7 with a 95% probability (42). The *CS*-coefficient is preferentially above 0.5, with a minimum requirement of 0.25 (42).

To test node strength or edge-weight differences, non-parametric bootstrapped difference tests were performed. Specifically, 1,000 bootstrapped CIs were constructed for the true node strength or edge-weight difference. If zero was included in the 95% CIs, there was no significant difference between two node or edge-weight strengths. All procedures were conducted using R package *bootnet and qgraph* (42).

### Associations Between Symptom Mean Levels, Variabilities, and Centralities

Spearman's rank-order correlations between symptom strengths and mean values were performed to examine whether the most central depressive symptoms were the most severe symptoms (23, 38). Then, Spearman's rank-order correlations between symptom centralities and SDs were performed to examine associations between strengths of depressive symptoms and their variabilities (22, 38).

### Gender Differences in Depressive Symptom Networks

To examine gender differences in depressive symptom networks (i.e., structure, edge strength, and global strength), a network comparison test (NCT) based on a 1000 permutation test was performed using the R package *NetworkComparisonTest* (39, 53). Edge-weight distributions of female and male networks were estimated for the comparison of the two network structures. Global strengths (i.e., the absolute sum of all edges of the networks) were also compared between female and male networks. Differences of each specific edge between female and male networks were estimated using Bonferroni corrections (53).

## Results

### Basic Demographic Characteristics

A total of 2,614 participants were invited to participate in this survey, of whom, 2.515 met inclusion criteria, and were included for analyses (women: 74.4%, 34.6 ± 10.7 years; men: 25.6%, 37.5 ± 11.2 years). A majority of participants completed post-secondary high education (i.e., Undergraduate/College or higher: 91.4%) and were married (62.8%) (Table 1).

**Table 1:** The socio-demographic information of all included samples.

**Variables**		**Total (*****N*** **= 2,515)**		**Female (*****N*** **= 1,870)**		**Male (*****N*** **= 645)**	
		**Mean/*N***	**SD/%**	**Mean/*N***	**SD/%**	**Mean/*N***	**SD/%**
Age (years)		35.4	10.9	34.6	10.7	37.5	11.2
Education	Junior high school	35	1.4	25	1.3	10	1.6
	High school	181	7.2	112	6.0	69	10.7
	Undergraduate/College	1926	76.6	1468	78.5	458	71.0
	Master/PHD	373	14.8	265	14.2	108	16.7
Marital status	Unmarried	820	32.6	629	33.6	191	29.6
	Married	1580	62.8	1153	61.7	427	66.2
	Divorced	102	4.1	77	4.1	25	3.9
	Widowed	9	0.4	7	0.4	3	0.3
	Others	4	0.2	4	0.2	0	0

*Mean/N, mean or sample number; SD, Standard deviation; SD/%, SD or percentage*.

Descriptive statistics for all depressive symptoms measured by the PHQ-9 are presented in Table 2. The mean and SD of the PHQ-9 item scores were 0.57 and 0.46, respectively. Suicide (P9) and Motor (P8) symptoms had the highest mean scores (0.90 and 0.74), while Fatigue (P4) and Anhedonia (P1) symptoms had the lowest mean ratings (0.33 and 0.40).

**Table 2:** Mean, standard deviation, minimum, maximum, skewness, kurtosis, and frequency of the PHQ-9 Symptoms (*N* = 2,515).

**PHQ-9 symptoms**	**Mean**	**SD**	**Min**	**Max**	**Skewness**	**Kurtosis**	**Absense (0) %**	**Presence (1–3) %**
1. Anhedonia	0.40	0.49	0	1	0.41	−1.83	0.40	0.60
2. Sad mood	0.48	0.50	0	1	0.06	−2.00	0.48	0.52
3. Sleep	0.45	0.50	0	1	0.22	−1.95	0.45	0.55
4. Fatigue	0.33	0.47	0	1	0.72	−1.48	0.33	0.67
5. Appetite	0.58	0.49	0	1	−0.33	−1.89	0.58	0.42
6. Guilt	0.60	0.49	0	1	−0.40	−1.84	0.60	0.40
7. Concentration	0.61	0.49	0	1	−0.44	−1.80	0.61	0.39
8. Motor	0.74	0.44	0	1	−1.12	−0.74	0.75	0.25
9. Suicide	0.90	0.30	0	1	−2.65	5.05	0.90	0.10

*SD, Standard deviation*.

*The reviewer JL declared a shared affiliation, with no collaboration, with the author TC at the time of the review*.

### Network Estimation and Strength Centrality

No item scores were lower than the 2.5 SD below the mean informativeness threshold (*M*_*SD*_=0.47±0.13) that indicated poor informativeness (38). Further, no item was found to be redundant with other items (37). Thus, all individual depressive symptoms were included for analysis.

**Figure 1:**
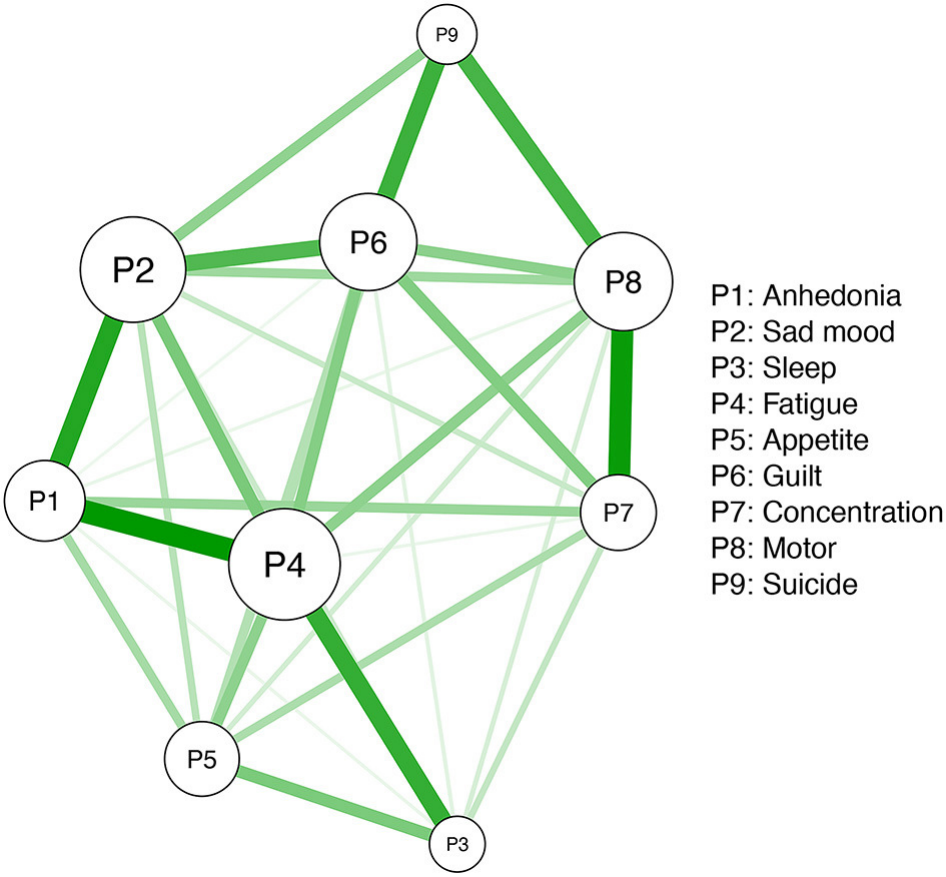
The estimated network structure of dichotomized PHQ-9 symptoms.

**Figure 2:**
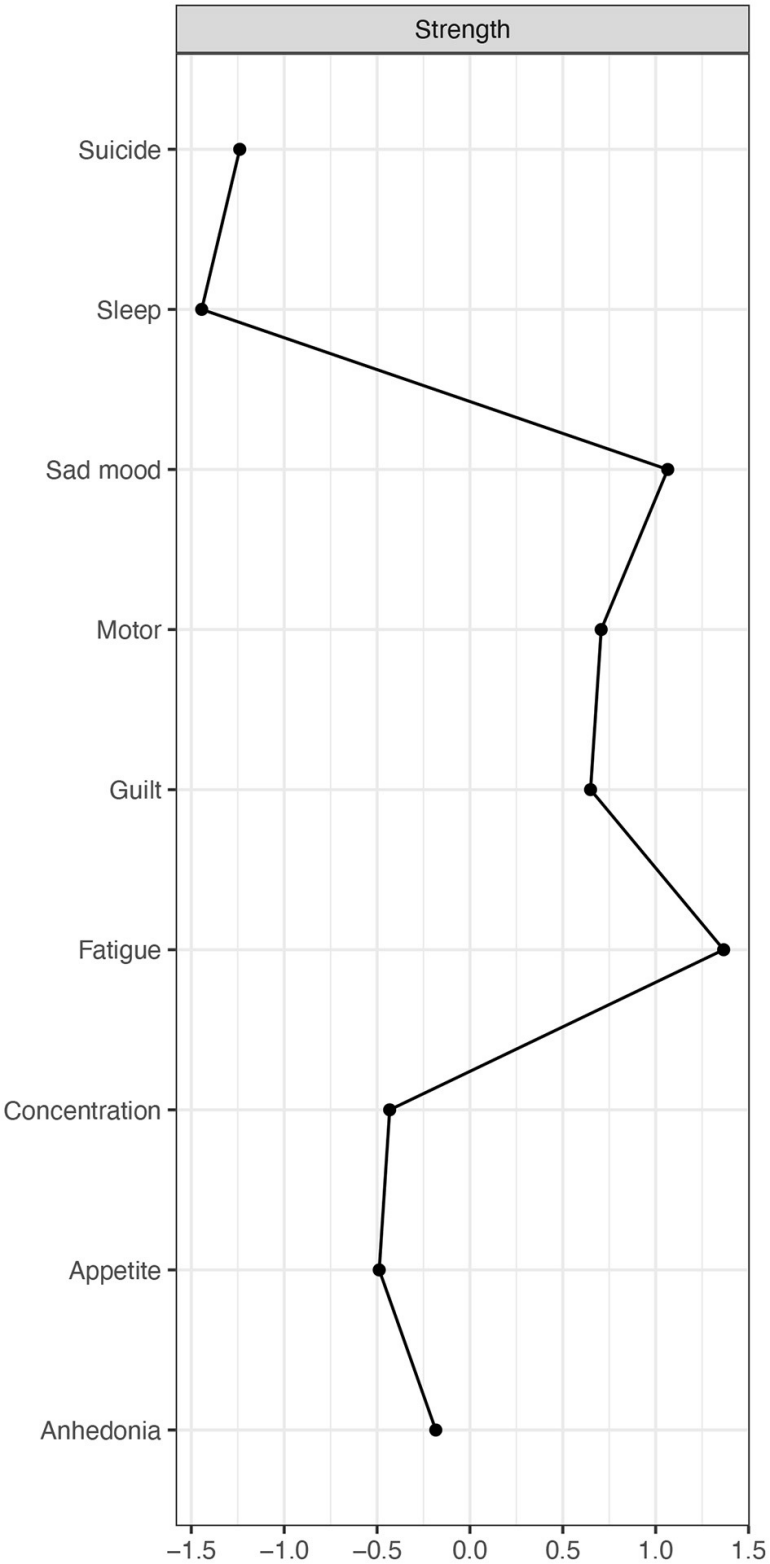
The node strength centrality in the PHQ-9 network.

The estimated symptom network based on the Ising model is displayed in Figure 1. Individual symptoms including Fatigue (P4), Sad mood (P2), and Guilt (P6) were significantly connected to the other symptoms. In addition, all depressive symptoms had positive correlations with each other (Supplementary Materials and Supplementary Table 1). Strengths of depressive symptoms are shown in Figure 2. Fatigue (P4) and Sad mood (P2) were the most central symptoms with the highest strengths, followed by Guilt (P6) and Motor disturbances (P8), while Sleep problems (P3) and Suicide (P9) symptoms had the lowest strength centrality (Figure 2).

### Network Accuracy and Stability

Accuracies of edges between pair nodes based on mean non-parametric bootstrapped CIs are shown in Supplementary Figure 1. Generally, observed edges in the network were consistent with the mean bootstrapped CIs. Larger edges displayed narrower CIs indicating more accuracy. This finding suggested the network was stable and robust (Supplementary Figure 2).

**Figure 3:**
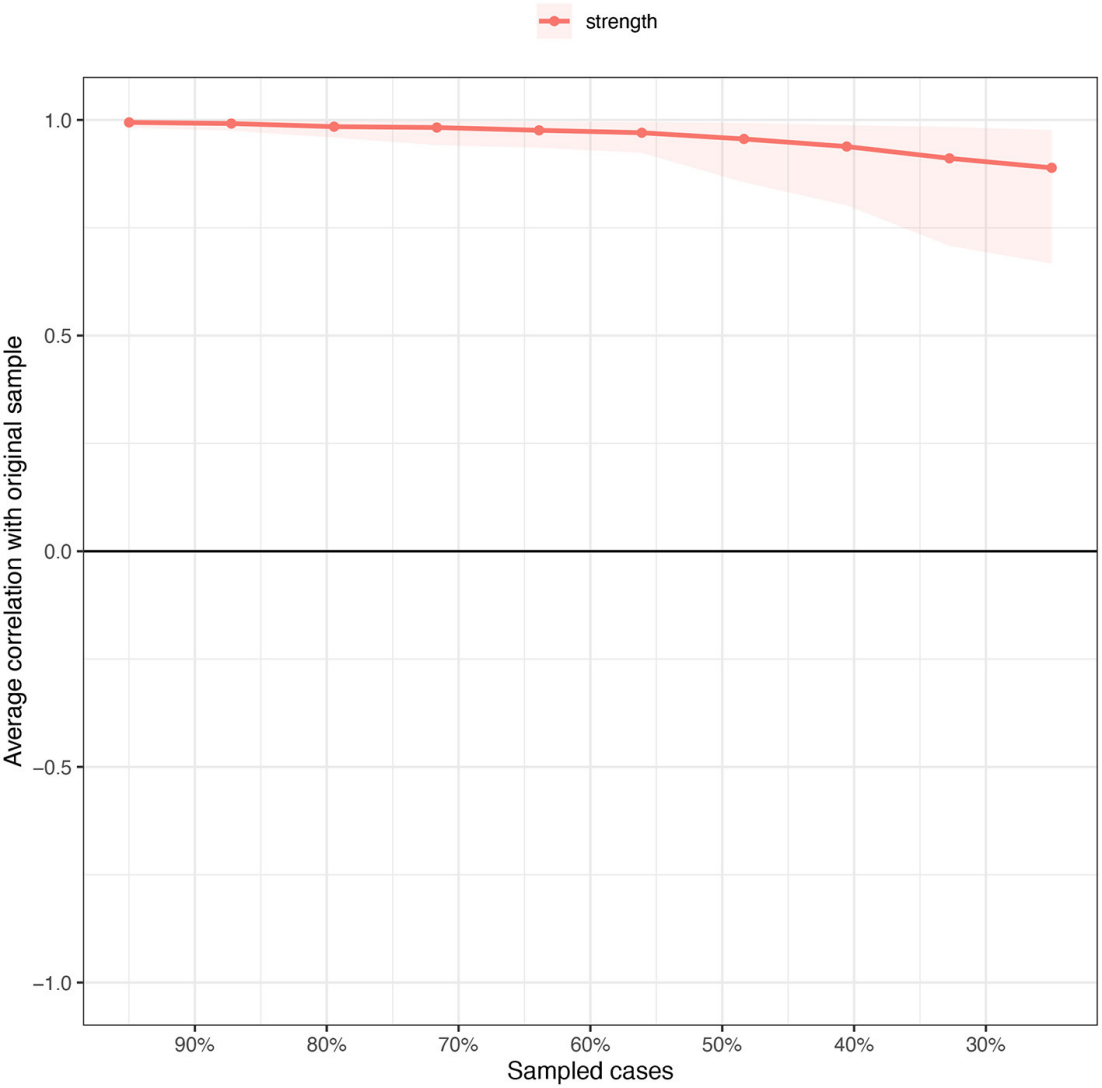
Stability of strength centrality (the average correlation with original sample) estimated by case dropping subset bootstrapped method.

Analyses of strength centrality reflected high stability (Figure 3). The CS-coefficient [CS (Cor = 0.7)] was 0.67 and indicated that the correlation coefficient between observed strength centrality of the subsets and that of the original sample still remained above 0.7 even after removing 67% of the cases.

**Figure 4:**
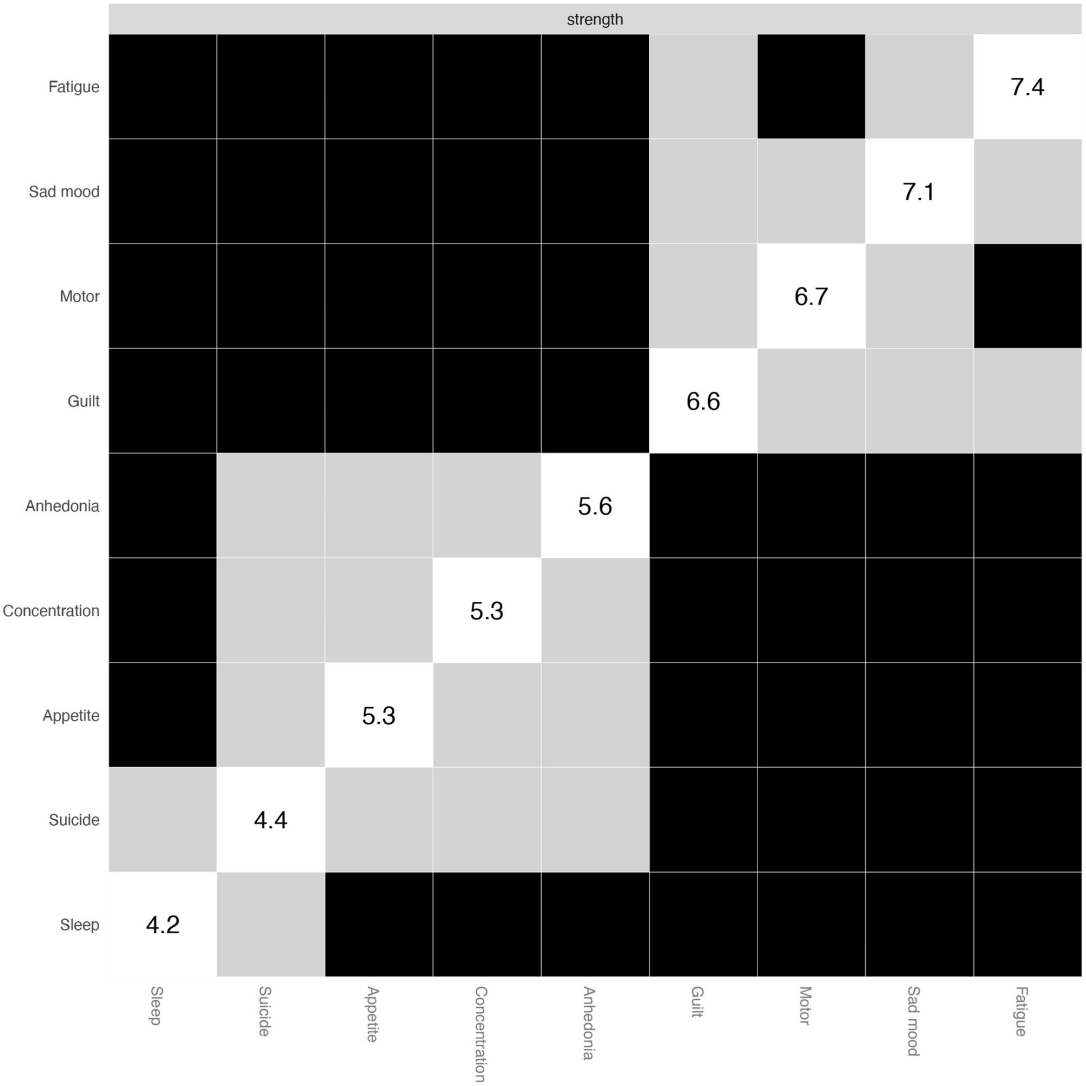
Non-parametric bootstrapped difference test for the node strength.

As shown in Figure 4, Fatigue (P4) and Sad mood (P2) symptoms had the highest strength centrality difference. In contrast, Sleep problems (P3) and Suicide (P9) symptoms had the lowest strength centrality difference. For the edge differences (see Supplementary Figure 2), a majority of edges, including those of Anhedonia (P2)-Fatigue (P4), Impaired concentration (P7)-Motor disturbances (P8), and Anhedonia (P1)-Sad mood (P2), were significantly different from the other edges.

### Associations Between Variabilities and Centralities

Of depressive symptoms, Suicide (P9), Impaired concentration (P7), Motor difficulties (P8), and Guilt (P6) symptoms had the highest mean values. However, mean values of individual depressive symptoms were not correlated with symptom strengths (*r*_s_ = −0.30, *p* = 0.43), or symptom variabilities (*r*_s_ = −0.47, *p* = 0.21); this pattern suggested that the centrality of depressive symptoms was not correlated with mean symptom levels and variabilities within the whole sample.

### Gender Comparisons of Depressive Symptom Networks

**Figure 5:**
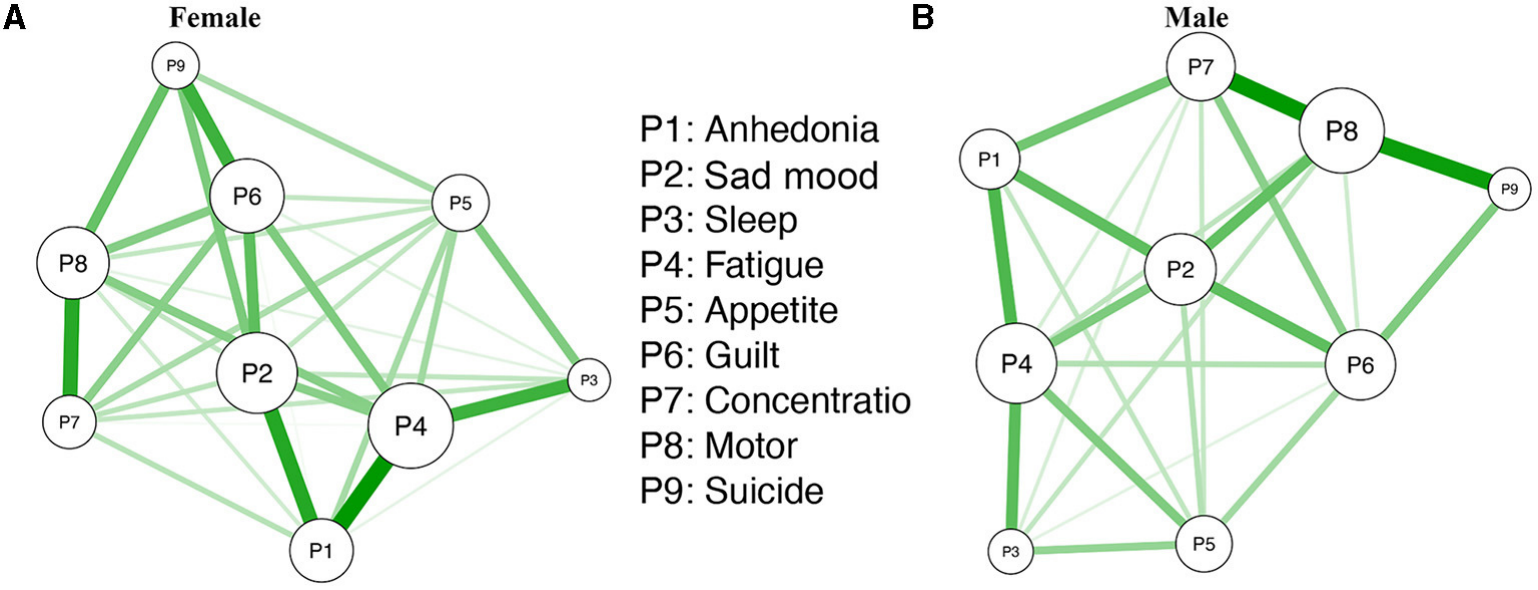
**(A,B)** The estimated dichotomized depressive network model for female and male participants.

Descriptive statistics for individual depression symptoms of women and men are presented in Supplementary Table 2. Figure 5 shows the estimated female (*n* = 1,870) and male (*n* = 645) networks. The two networks did not have significant differences in either network structure (maximum difference=0.11, *p* = 0.44) or network centralities (global strength difference=0.04, female vs. male: 3.78 vs. 3.83, *p* = 0.51). In analyses of gender differences in individual edge levels based on Bonferroni-Holm corrections, a majority of the edges did not differ significantly between women and men. However, there were two exceptions: (1) Anhedonia (P1)- Impaired concentration (P7) (female vs. male: 0.59 vs. 1.29, the difference *p* = 0.039), and (2) Sad mood (P2)-Motor difficulties (P8) (female vs. male: 0.49 vs. 1.44, the difference *p* = 0.002), both of which suggest that different treatment priorities should be considered due to different clinical features between women and men.

### Estimated Depressive Symptom Network Using the EBICglasso Model

As the optimal approach modeling the trichotomous items is still in debate (23), we also performed a network estimation using EBICglasso model to evaluate the robustness of network results. As shown in Supplementary Figure 4, findings for strength were similar to those observed using the Ising model (*r* = 0.93, 95%CI: 0.99–0.71) and edges (*r* = 0.93, 95%CI: 0.89–0.95).

### Depressive Symptom Network After Controlling for Age, Gender, Marital Status, and Education

We re-estimated the model using the mgm model after controlling for age, gender, marital status, and education. Results were consistent with the primary results based on the Ising model with no covariates (Supplementary Figure 5). Consistencies for both strengths and edges were very high (*r* = 0.92, 95%CI: 0.65–0.98; *r* = 0.92, 95% CI: 0.88–0.95).

## Discussion

This was the first study to explore the structure of the depression symptom network among Wuhan residents shortly after the COVID-19 epidemic peak. Fatigue had the highest centrality, followed by Sad mood, Guilt and Motor difficulties. Fatigue, usually defined as a loss of energy (54, 55), feeling tired (56), or exhaustion (57), is particularly relevant to anhedonia in depression and other psychiatric disorders (58, 59). As expected, Sad mood also emerged as one of the most central symptoms, replicating previous findings (60), and underscoring its role as a hallmark symptom of depression.

Guilt (i.e., regret, feeling like a disappointment to oneself or others based on the PHQ-9) (34, 35), refers to personal negative assessment of one's behaviors, and usually evolves from caring, cooperative, and harm avoidance motives (61, 62). In this network analysis of depression, Guilt was strongly associated with not only Sad mood, but also with Suicide ideation, which is consistent with previous findings (63, 64). For instance, one study (63) found that persons who experienced serious trauma with feeling of worthlessness were more likely to attempt suicide. Similarly, Wakefield and Schmitz (64) found that guilt was the only significant predictor of increased risk of post remission suicide attempts among depressive symptoms. In addition, a more recent network analysis (65) revealed that self-worthlessness was the most central symptom in depression model. In the context of living at the epicenter of China's pandemic, we speculate that depressive experiences of Wuhan residents are characterized more strongly by feelings of guilt related to witnessing illness or death from the COVID-19 outbreak or, possibly, from surviving the epidemic while others have perished as a result of its spread (1, 66, 67).

Motor difficulties were another central symptom in this depression network model, which confirms the notion that psychomotor problems are among the most important individual symptoms in depression (68–72). This could be partly attributed to disrupted structural and functional coupling between different brain networks (73, 74). For example, Ge and colleagues found an attenuated positive correlation between the right ventral-posterior insular structural covariance and motor and psychomotor performance among depressed patients compared to healthy controls (73). Further, compared with healthy controls, both lower within-putamen functional connectivity (75) and decreased cerebral blood flow of right primary motor cortex (76) are associated with more severe psychomotor retardation in depressed patients (75, 76). Additionally, certain public health measures including lockdown and social distancing during the COVID-19 pandemic could contribute to reduced activities (71, 77), which, in turn, exacerbated psychomotor retardation (1). Furthermore, we also found a stronger edge between Motor difficulties and Suicide ideation, which is consistent with an earlier finding Malhi et al. (78) that both difficulties in initiating activities and suicidal ideation are the strongest predictors of severe depression.

There was no correlation between mean values of individual depressive symptoms and their strength centrality and variability. For example, similar to previous findings (60), Suicide ideation had the lowest centrality strength in the network model, but also had the highest mean level among individual depressive symptoms. Fear of infection, social isolation, uncertainty and economic loss associated with COVID-19 could lead to various psychological problems, which finally increased suicide ideation, especially for individuals who reside in Wuhan, the epicenter and those whose family members or friends died or were infected with COVID-19 during the pandemic (8, 79–82). This overall pattern is consistent with findings from clinical research. A previous study found that imipramine and Mindfulness-Based Cognitive Therapy both significantly improved mean levels of depressive symptoms but failed to change the dynamic depressive symptom network structure over time (83). In contrast, psychosocial inventions targeting central depressive symptoms (i.e., the symptoms with high strength centrality) may be much more effective; these interventions improve the severity of central symptoms themselves in addition to reducing the severity of other symptoms in the network that have connections with central symptoms (26). Thus, apart from the treatments targeting specific individual symptoms with high mean levels such as suicide ideation, interventions targeting central symptoms, i.e., Fatigue, Sad mood, Guilt and Motor difficulties, should be considered to reduce overall depression severity and increase treatment efficacy based on findings from network analysis.

Gender comparisons of depressive symptom networks indicated similar overall network structures and global strengths between women and men, consistent with a recent finding (84). However, in this study, male participants had higher edge weights in Anhedonia (P1)-impaired concentration (P7), and in Sad mood (P2)-Motor difficulties (P8) nodes compared to women, findings that are potentially due to gender differences in the psychopathology of depression (85–89). Previous studies (88) found that men tended to be depressed with functional limitations, and were more likely to handle depression by increasing physical activities; in contrast, women tended to handle it through emotional release or religion (86). However, physical activities were greatly limited due to quarantine measures during the COVID-19 pandemic, which might have worsened sad mood in men. The gender differences were novel, therefore replications are needed to determine whether these results were specific to this sample or reflective of general gender differences.

Strengths of this study included its large sample size, use of different network analysis models (e.g., Ising and EBICglasso) to assess consistency of overall results, and replications of initial sample results even after statistically controlling for socio-demographic correlates of depression. Several limitations should be noted. First, due to the cross-sectional study design, causal relationships between these depressive symptoms and dynamic changes in symptom networks over time could not be established. Second, this study was conducted at China's epicenter shortly after the COVID-19 epidemic peak. Therefore, network model findings may not be generalized to different phrase of the pandemic or sites that were relatively unaffected by COVID-19. In addition, the education level of participants was skewed, as majority received college education. However, after controlling for education as well as age, gender, and marital status as covariates, results did not change significantly. Finally, the study sample was drawn from the general community which limits the generalizability of findings to clinical samples.

In conclusion, the network analysis indicated that Fatigue, Sad mood, Guilt and Motor impairments are central symptoms in the depressive network of women and men in a community sample during the months that followed the COVID-19 peak at the epicenter of China's epidemic. As such, these symptoms should be prioritized as the targets in treatment and prevention interventions for depression among adult residents in Wuhan in the later stage of the COVID-19 pandemic.

## Data Availability Statement

The Ethics Committee of Beijing Anding Hospital that approved the study prohibits the authors from making the research dataset of clinical studies publicly available. Readers and all interested researchers may contact Dr. Y-TX (Email address: xyutly@gmail.com) for details. Dr. Xiang would apply to the Ethics Committee of Beijing Anding Hospital for the release of the data.

## Ethics Statement

The studies involving human participants were reviewed and approved by Beijing Anding Hospital. The patients/participants provided their written informed consent to participate in this study.

## Author Contributions

S-FZ, BY, Y-FZ, and Y-TX: study design. NZ, WL, SS, S-FZ, and BY: data collection, analysis, and interpretation. NZ, TC, and Y-TX: drafting of the manuscript. TJ: critical revision of the manuscript. All authors approval of the final version for publication.

## Funding

The study was supported by the National Science and Technology Major Project for investigational new drug (2018ZX09201-014), the Beijing Municipal Science & Technology Commission (No. Z181100001518005), the University of Macau (MYRG2019-00066-FHS), the Fundamental Research Funds for the Central Universities (2020YJ065), and the Key Realm R&D Program of Guangdong Province (2019B030335001).

## Conflict of Interest

The authors declare that the research was conducted in the absence of any commercial or financial relationships that could be construed as a potential conflict of interest. The reviewer JL declared a shared affiliation, with no collaboration, with the author TC at the time of the review.

## Publisher's Note

All claims expressed in this article are solely those of the authors and do not necessarily represent those of their affiliated organizations, or those of the publisher, the editors and the reviewers. Any product that may be evaluated in this article, or claim that may be made by its manufacturer, is not guaranteed or endorsed by the publisher.
