# Supplementary material for: Network Analysis of Depressive Symptoms Among Residents of Wuhan in the Later Stage of the COVID-19 Pandemic
Source: Front Psychiatry. 2021 Sep 30;12:735973. doi: 10.3389/fpsyt.2021.735973 (PMC8514718; doi:10.3389/fpsyt.2021.735973)
Supplement: Supplementary file 1 [file Data_Sheet_1.docx]

Supplementary Material

# Supplementary Tables

**Table S1. Weighted adjacency matrix**

|  | **Anhedonia** | **Depression** | **Sleep** | **Fatigue** | **Appetite** | **Guilt** | **Concentration** | **Motor** | **Suicide** |
| --- | --- | --- | --- | --- | --- | --- | --- | --- | --- |
| **Anhedonia** | 0.00 | 1.72 | 0.16 | 1.99 | 0.68 | 0.13 | 0.78 | 0.16 | 0.00 |
| **Sad mood** | 1.72 | 0.00 | 0.40 | 1.03 | 0.55 | 1.36 | 0.42 | 0.75 | 0.86 |
| **Sleep** | 0.16 | 0.40 | 0.00 | 1.58 | 1.02 | 0.22 | 0.46 | 0.31 | 0.00 |
| **Fatigue** | 1.99 | 1.03 | 1.58 | 0.00 | 0.87 | 0.94 | 0.16 | 0.87 | 0.00 |
| **Appetite** | 0.68 | 0.55 | 1.02 | 0.87 | 0.00 | 0.58 | 0.64 | 0.34 | 0.58 |
| **Guilt** | 0.13 | 1.36 | 0.22 | 0.94 | 0.58 | 0.00 | 0.96 | 0.89 | 1.51 |
| **Concentration** | 0.78 | 0.42 | 0.46 | 0.16 | 0.64 | 0.96 | 0.00 | 1.91 | 0.00 |
| **Motor** | 0.16 | 0.75 | 0.31 | 0.87 | 0.34 | 0.89 | 1.91 | 0.00 | 1.44 |
| **Suicide** | 0.00 | 0.86 | 0.00 | 0.00 | 0.58 | 1.51 | 0.00 | 1.44 | 0.00 |

Note: The weighted adjacency matrix represents the weight of direct edges between node

**Table S2. Mean, standard deviation, skewness, and kurtosis, and frequency of depressive symptoms as measured using the PHQ-9 by gender.**

|  | **Female (n=1870)** | | | | | | **Male (n=668)** | | | | | |
| --- | --- | --- | --- | --- | --- | --- | --- | --- | --- | --- | --- | --- |
| **Symptoms** | **mean** | **sd** | **skewness** | **kurtosis** | **Absense (0, %)** | **Presence (1-3, %)** | **mean** | **sd** | **skewness** | **kurtosis** | **Absense (0, %)** | **Presence (1-3, %)** |
| **1. Anhedonia** | 0.39 | 0.49 | 0.45 | -1.80 | 61.0 | 39.0 | 0.43 | 0.50 | 0.28 | -1.92 | 57.1 | 42.9 |
| **2. Sad mood** | 0.48 | 0.50 | 0.09 | -1.99 | 52.4 | 47.6 | 0.51 | 0.50 | -0.03 | -2.00 | 49.3 | 50.7 |
| **3. Sleep** | 0.44 | 0.50 | 0.26 | -1.93 | 56.5 | 43.5 | 0.48 | 0.50 | 0.09 | -2.00 | 52.2 | 47.8 |
| **4. Fatigue** | 0.32 | 0.47 | 0.79 | -1.38 | 68.3 | 31.7 | 0.37 | 0.48 | 0.55 | -1.70 | 63.3 | 36.7 |
| **5. Appetite** | 0.57 | 0.49 | -0.30 | -1.91 | 42.6 | 57.4 | 0.60 | 0.49 | -0.41 | -1.83 | 39.8 | 60.2 |
| **6. Guilt** | 0.60 | 0.49 | -0.42 | -1.83 | 39.7 | 60.3 | 0.58 | 0.49 | -0.34 | -1.89 | 41.7 | 58.3 |
| **7. Concentration** | 0.61 | 0.49 | -0.46 | -1.79 | 38.9 | 61.1 | 0.60 | 0.49 | -0.41 | -1.84 | 40.0 | 60.0 |
| **8. Motor** | 0.76 | 0.43 | -1.20 | -0.57 | 24.3 | 75.7 | 0.71 | 0.45 | -0.92 | -1.15 | 29.0 | 71.0 |
| **9. Suicide** | 0.90 | 0.30 | -2.63 | 4.90 | 10.2 | 89.8 | 0.90 | 0.30 | -2.73 | 5.48 | 9.6 | 90.4 |

**SD: standardized deviation**

# Supplementary Figures

**Figure S1 Nonparametric bootstrapped difference test for edge weights of 9 PHQ symptoms.** The gray boxes indicate individual edge is not significantly different from others, while black boxes represent statistically significant difference (α = 0.05). The saturation of the diagonal blue color represents the magnitude of the positive estimated edge.

**Figure S2. The nonparametric bootstrapped confidence intervals of edge-weights for PHQ-9 symptoms.** The black dots indicate the edge weights ranked from the highest to the lowest. The *red line* indicates the sample values and the gray area the bootstrapped CIs. The wider gray area represents the lower accuracy.

**Figure S3. The standardized centrality indices of depressive symptoms for female and male participants.**

**
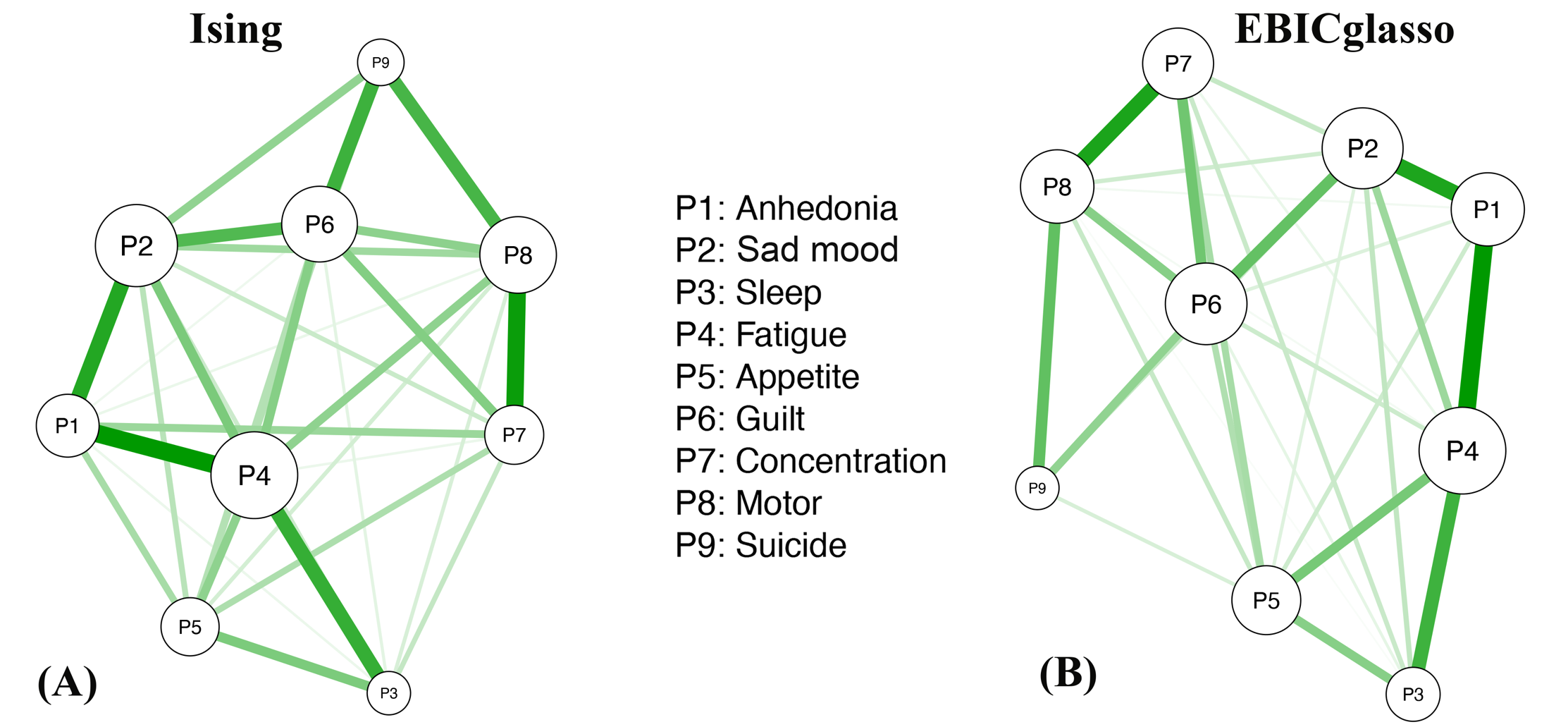
**

**Figure S4. The estimated depressive networks using Ising model (A) and EBICglasso model (B).**

**The different size circles correspond to different strengths of the nodes.**

**
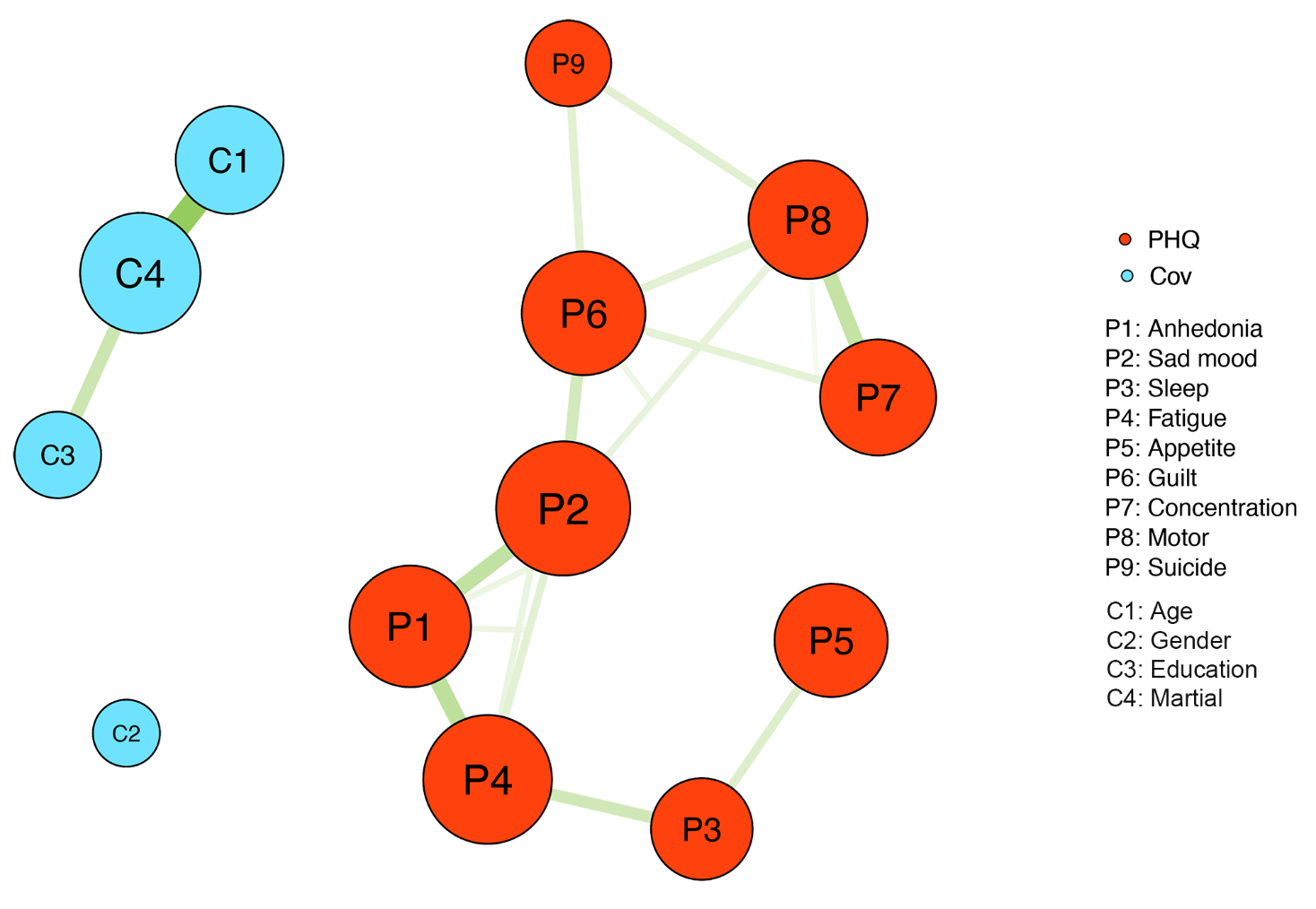
**

**Figure S5. The estimated depressive network by adding age, gender, education and marital status as covariates. The different size circles correspond to different strengths of the nodes.**
